# The role of recombination in the emergence of a complex and dynamic HIV epidemic

**DOI:** 10.1186/1742-4690-7-25

**Published:** 2010-03-23

**Authors:** Ming Zhang, Brian Foley, Anne-Kathrin Schultz, Jennifer P Macke, Ingo Bulla, Mario Stanke, Burkhard Morgenstern, Bette Korber, Thomas Leitner

**Affiliations:** 1 Theoretical Biology & Biophysics, Los Alamos National Laboratory, Los Alamos, NM 87545, USA; 2 Center for Nonlinear Studies, Los Alamos National Laboratory, Los Alamos, NM 87545, USA; 3 Institut für Mikrobiologie und Genetik, Abteilung Bioinformatik, Goldschmidtstraße 1, 37077 Göttingen, Germany; 4 The Santa Fe Institute, Santa Fe, NM 87501, USA

## Abstract

**Background:**

Inter-subtype recombinants dominate the HIV epidemics in three geographical regions. To better understand the role of HIV recombinants in shaping the current HIV epidemic, we here present the results of a large-scale subtyping analysis of 9435 HIV-1 sequences that involve subtypes A, B, C, G, F and the epidemiologically important recombinants derived from three continents.

**Results:**

The circulating recombinant form CRF02_AG, common in West Central Africa, appears to result from recombination events that occurred early in the divergence between subtypes A and G, followed by additional recent recombination events that contribute to the breakpoint pattern defining the current recombinant lineage. This finding also corrects a recent claim that G is a recombinant and a descendant of CRF02, which was suggested to be a pure subtype. The BC and BF recombinants in China and South America, respectively, are derived from recent recombination between contemporary parental lineages. Shared breakpoints in South America BF recombinants indicate that the HIV-1 epidemics in Argentina and Brazil are not independent. Therefore, the contemporary HIV-1 epidemic has recombinant lineages of both ancient and more recent origins.

**Conclusions:**

Taken together, we show that these recombinant lineages, which are highly prevalent in the current HIV epidemic, are a mixture of ancient and recent recombination. The HIV pandemic is moving towards having increasing complexity and higher prevalence of recombinant forms, sometimes existing as "families" of related forms. We find that the classification of some CRF designations need to be revised as a consequence of (1) an estimated > 5% error in the original subtype assignments deposited in the Los Alamos sequence database; (2) an increasing number of CRFs are defined while they do not readily fit into groupings for molecular epidemiology and vaccine design; and (3) a dynamic HIV epidemic context.

## Background

Retroviral recombination introduces rapid, large genetic alternations [1-3], and can repair genome damage [4,5]. Recombination is a major force in HIV evolution, occurring at an estimated rate of at least 2.8 crossovers per genome per cycle [6]. Recently the effective recombination rate, i.e., the product of super-infection and crossovers, was estimated to be on a similar frequency as the nucleotide substitution rate within patients (1.4 × 10^-5 ^recombinations per site and generation) [7]. Recombination between HIV-1 subtypes may result in establishing epidemiologically important founder strains. Recombinant lineages can contribute to secondary recombination events, leaving traces of ever more complex diversity patterns and confounding classical phylogenetics [8]. Within a single host, recombination may produce variants resistant to HIV-1 specific drugs and immune pressure [9-12].

At least 20% of HIV-1 isolates sequenced worldwide are inter-subtype recombinants [13-16]. These recombinants are classified into two categories, CRFs (circulating recombinant forms) and URFs (unique recombinant forms), referring to recombinants that have established recurrent and transmitted forms in populations, and to those only identified in one individual, respectively [17]. Currently, more than 40 CRFs and 100 URFs have been identified worldwide http://www.hiv.lanl.gov. Globally, these numbers are increasing as a result of multiple subtypes (and recombinants) in local epidemics, thus providing the biological context for inter-subtype recombination. The number of detected recombinants is also increasing due to improved technology allowing rapid large-scale genome sequencing and the availability of more advanced recombination detection software.

It is estimated that CRF02_AG, a CRF derived from subtype A and G, has caused at least 9 million infections worldwide [18]. First identified in Nigeria in 1994 [19], it is the most prevalent strain in West and West Central Africa. In Cameroon, where the original HIV-1 M group zoonotic transmissions are believed to have taken place [20,21], CRF02 was already prevalent in the early 1990s [22], and it is currently the dominant lineage in this part of the world [20,23]. It is possible that CRF02's high prevalence in Africa is explained by its long presence in the epidemic. Comparing the genetic diversity within CRF02 to that occurring within pure subtypes, Carr *et al*. suggested that CRF02 may be as old as the pure subtypes [24]. A recent study proposed the idea that CRF02_AG was a parent of subtype G [25], rather than subtype A and G being parental strains of CRF02.

CRF07_BC and CRF08_BC are the most common BC recombinants. CRF07 was first identified in the Xinjiang province of China in 1997 [18,21,26,27], and it is believed to have migrated to Xinjiang along a northern drug trafficking route [26,28]. CRF08 is a predominant subtype among intravenous drug users (IDUs) in Guangxi and the east part of the Yunnan province in China [26,29]. Both CRFs presumably originated in Yunnan where subtypes B and C were co-circulating in the early 1990s [30-33], or in Myanmar and then imported from there into China [34-37]. It has not been established whether other BC recombinants in Myanmar and China are epidemiologically linked to the CRF07 and CRF08 HIV-1 epidemic in Southern China [38,39].

BF recombinants in South America are dominated by a large number of recombinants with unique breakpoint patterns, URFs http://www.hiv.lanl.gov, geography page.  The BF epidemic in this region is characterized by two genetic centers. One is represented by CRF12_BF and related genomes that are more frequently found in Argentina; and the other by CRF28_BF, CRF29_BF and a collection of BF URFs that have been found in Brazil [40]. The origin of BF recombinants in South America is not clear, but it appears that at least one of the main introductory routes of HIV-1 into South America was through Brazil [41].

Accurate virus genotyping and recombination identification techniques are important for many reasons, including epidemiological tracking, targeting vaccines to regional epidemics, understanding the evolutionary trajectory of the virus, and defining potential phenotypic differences in different subtypes or inter-subtype recombinants [42]. Here we report results from a large-scale subtyping study of 9435 sequences that includes subtypes A, B, C, G, F, and CRFs and URFs exclusively composed of subtypes A and G, or B and C, or B and F. These sequences include all circulating recombinant forms dominating three epidemically important regions: West and West Central Africa, southern China, and South America. A series of detailed analyses were performed to ensure genotyping quality. Therefore, our analyses can provide a more comprehensive image of the current HIV epidemics in these three geographic regions. We demonstrate strong evidence that the recombinant lineages that are highly prevalent in the current HIV epidemic are a mixture of ancient and recent recombinant lineages. The dynamic HIV epidemic is moving toward having increasing complexity and higher prevalence of recombinant forms. Finally we suggest that a revision of some CRFs may be needed.

## Results

### Genotyping results and comparisons to the original subtype assignments suggest that a revision of some CRF designations may be needed

In total, we genotyped 9435 near full-length and sequence fragments obtained from the Los Alamos HIV sequence database and compared our results to the subtype assignments derived from the original literature (Table 1). Overall, 4.9% of the subtype assignments were inconsistent. The number of inconsistent assignments were unevenly distributed among sequence lengths such that shorter fragments more often than near full-length sequences disagreed: Among BC recombinants, 59.6% of the sequence fragments were assigned differently in our results as compared to the original author assignments (Table 1, BC column). This difference is, however, not as dramatic as it may seem. For example, all literature-assigned CRF08 sequence fragments were assigned as pure subtypes in our results - in one case subtype B and in the rest subtype C. Given that it is difficult to resolve the subtype in un-sequenced regions outside a sequence fragment, it becomes a philosophical nomenclature question of which assignment is best for sequence fragments embedded in a genomic region that is spanned by just one subtype constituting the locally prevalent CRF, i.e., to assign a sequence fragment with "CRF08", "C" or "B". When the HIV nomenclature procedures were first outlined [17], for the sake of consistency, there was a decision to use the subtype designation when a fragment was too short to span known breakpoints for CRFs. Thus the convention we use assigns the sequences based on the available information, e.g., a C fragment should be assigned as "C" even if it is suggested that CRF08 is known to be common in the geographic region where the sequence was isolated and even if the C is closer to the C in CRF08 rather than to a pure C (note also that this distinction often cannot be made with confidence). Finally, unless the whole genome is sequenced one cannot know what the classification is in uninvestigated regions. Thus, in agreement with the original HIV nomenclature proposal we have assigned fragments to their closest subtypes (or CRF) but not guessed what the rest of the genome is.

**Table 1:** Comparison of subtype assignments (jpHMM results versus current database assignment that is based on the original literature)

			AG set				BC set									
**Num of sequences**			**Full length (world)** **N = 140**				**Full length (world)** **N = 509**					**Fragments (Asia)** **N = 4413**				

**Database subtype**			**A**	**G**	**02**	**AG**	**B**	**C**	**07**	**08**	**BC**	**B**	**C**	**07**	**08**	**BC**

**Num of sequences**			72	12	48	8	152	334	7	4	12	3133	1048	17	171	44

**Num of problematic sequences^1^**			1	0	2	0	15	12	0	0	3	0	0	0	0	0

**Num of discordant sequences^2^**			0	0	1	0	2	0	0	0	2	24	6	6	102	27

			**BF set**													

**Num of sequences**			**Full length (world)** **N = 220**							**Fragments (S. America)** **N = 4153**						

**Database subtype**	**B**		**F**		**12**	**17**	**28**	**29**	**BF**	**B**	**F**	**12**	**17**	**28**	**29**	**BF**

**Num of sequences**	152		12		11	2	3	4	36	3070	242	261	0	0	0	580

**Num of problematic sequences^1^**	15		0		0	0	0	0	0	0	0	0	0	0	0	0

**Num of discordant sequences^2^**	2		2		6	2	1	1	1	74	19	31	0	0	0	107

1. Problematic sequences are those that could not be unequivocally assigned. They meet one of the following criteria: 1) Contain an unusually high content of IUPAC code N (defined as > 100 continuous Ns, or > 7% N for sequences of length < 1000 nt, or > 5% N for sequences of length 1000-2999, or > 3% N for sequences of length 3000 or above); 2) Contain an artifactual deletion of > 100 nt.

2. Classification of the sequences was compared between the database assignments (of which the majority were extracted from the literature) and the jpHMM predictions.

**Figure 1:**
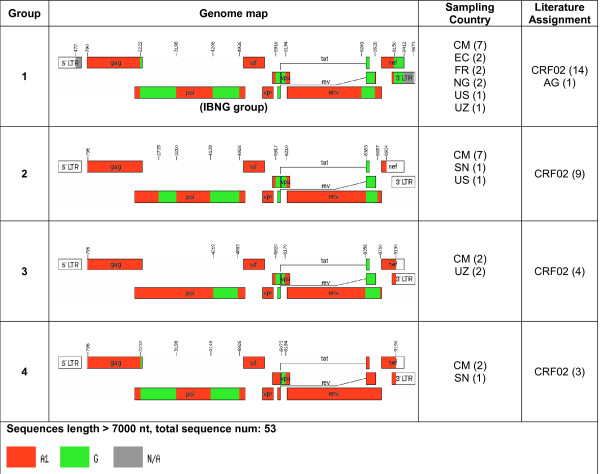
**Genome maps of all near full-length sequences composed exclusively of subtypes A and G**. AG recombinants were classified into 4 groups and 22 URFs. A group is defined as a set of sequences (>1) that have identical breakpoints. The genomic compositions and breakpoint positions were computed by the jpHMM program as described in Materials and Methods. The 22 URFs were originally assigned as CRF02 in 15 cases and different AG recombinants in 7 cases. They were sampled in CM (n = 10), GH (3), NG (2), SN (2), BE (1), CD (1), KE (1), SE (1), and US (1). "Sampling Country" is abbreviated by ISO standard 2-letter codes [AR: Argentina. BE: Belgium, BO: Bolivia, BR: Brazil, CD: Dem Rep of the Congo, CL: Chile, CM: Cameroon, CN: China, EC: Ecuador, ES: Spain, FR: France, GH: Ghana, KE: Kenya, MM: Myanmar. NG: Nigeria, SE: Sweden, SN: Senegal, US: United States, UY: Uruguay, UZ: Uzbekistan, VE: Venezuela.] "Literature Assignment" refers to the legacy HIV database/literature-assigned subtypes. In parenthesis are the numbers of sequences.

**Figure 2:**
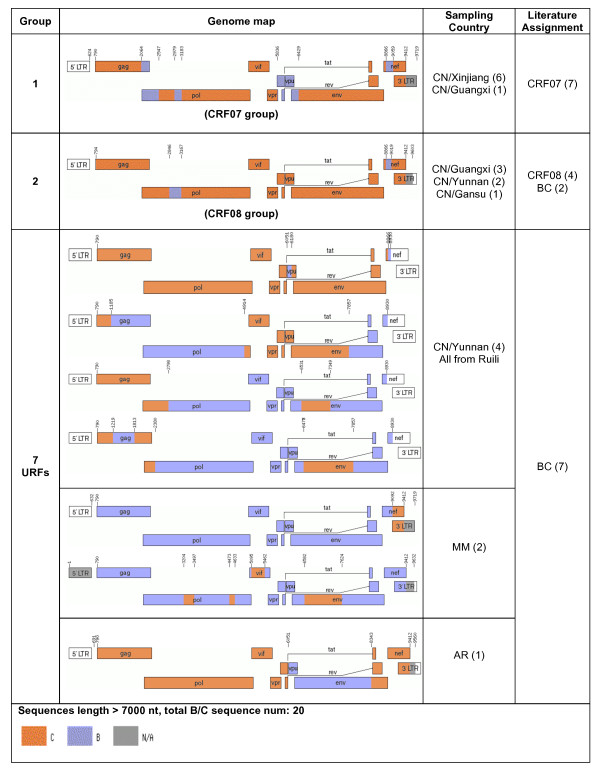
**Genome maps of all near full-length sequences composed exclusively of subtypes B and C**. BC recombinants were classified into 2 groups and 7 URFs. Group definitions and country codes are as in Fig 1.

**Figure 3:**
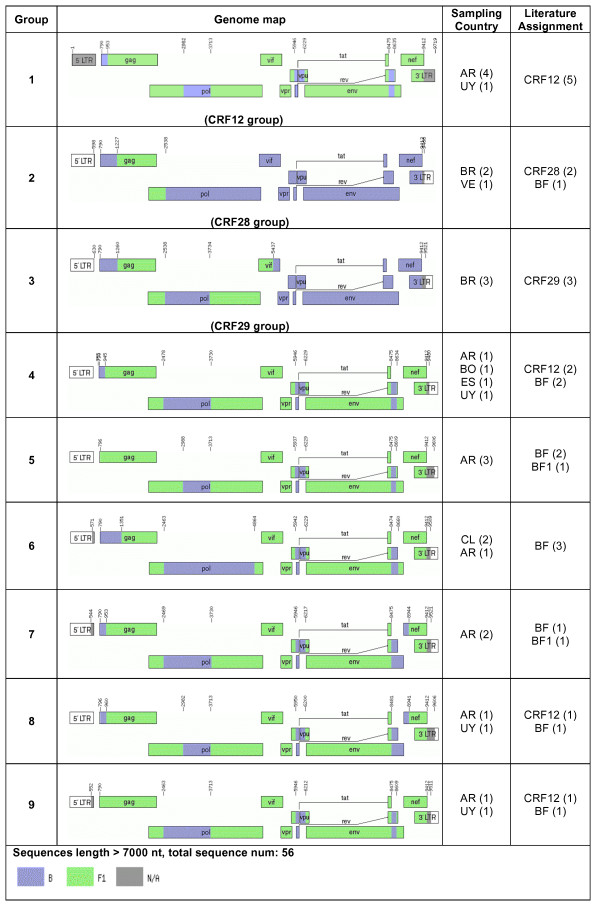
**Genome maps of all near full-length sequences composed exclusively of subtypes B and F**. BF recombinants were classified into 9 groups and 29 URFs. Group definitions and country codes are as in Fig 1. The 29 URFs were originally assigned as CRF 12 (n = 2), CRF17 (2), CRF28 (1), CRF29 (1), and different BF recombinants in 23 cases. They were sampled in BR (n = 18), AR (9), CL (1), and ES (1).

Next, all near full-length AG, BC, and BF recombinants were grouped into common groups if the sequences had similar genomic structures and breakpoints (Figs. 1, 2, and 3). Our results suggested that revisions of some CRF designations may be needed. For instance, some database-assigned BF CRF sequences in this analysis appear to be unique BF URFs with atypical breakpoints (Fig. 3). In case of CRF17, two previous sequences (accession number: AY037275 and AY037277) were assigned as CRF17 prototype sequences. They were, however, epidemiologically linked [43]. Another 7 sequences of CRF17 (mostly unpublished) have now been made available. These sequences consist of related, but not identical, recombinant forms that could be described as a "family" of recombinants (see further discussion on this topic).

The CRF and URF sequences described below refer to the sequences confirmed by our jpHMM genotyping results.

### CRF02 is a recombinant lineage with both early and more recent recombination events involving subtypes A1 and G

**Figure 4:**
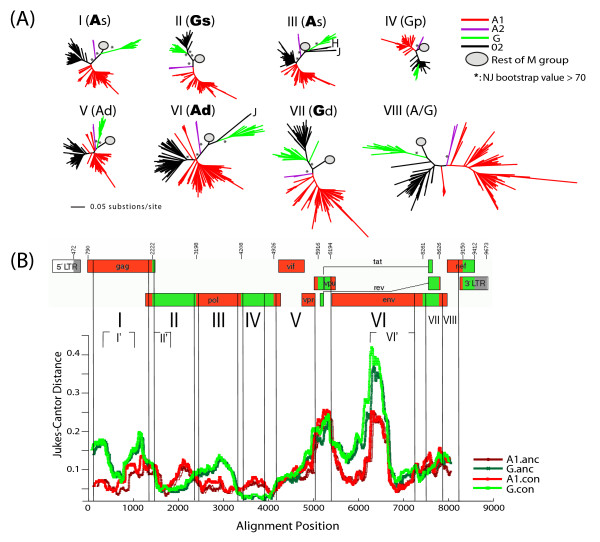
**CRF02 is a recombinant derived from old and contemporary subtypes A and G**. (A) Maximum Likelihood trees of consensus sub-regions delimited by breakpoints shared by most CRF02 and AG recombinant sequences. Bootstrap support values for clustering are shown. The relationship between CRF02 and subtypes A and G inferred from the ML results is defined as: CRF02 is a sibling of subtype A (As), sibling of G (Gs), parent of G (Gp), descendent of A (Ad), descendent of G (Gd), a mixture between A and G but do not cluster with either A or G (A/G). The relationships supported by ≥ 70% bootstrap values are in bold, otherwise in plain font. (B) The consensus sub-regions (I through VIII) were mapped onto the HXB2 genome. Also shown here is the RIP result for assessing CRF02's similarity to the ML-derived-ancestral and contemporary-consensus sequences of subtypes A and G.

To examine the evolutionary relationships among recombinants that are exclusively composed of subtypes A and G, as well as their relationships with all sequences of pure subtypes A and G, we performed phylogenetic analyses in eight sub-regions (Fig. 4, Regions I-VIII) delimited by the shared breakpoints of most full-length AG sequences depicted in Figure 1. IBNG is considered a prototype strain of CRF02, and was found representative of the most common AG-lineage (Group 1, Fig. 1). Other sequences, however, did not cluster with the same subtype as IBNG in all studied genomic regions, indicating subsequent secondary recombination events with other A and G viruses. Interestingly, some genomic regions suggest that CRF02 is an old recombinant derived from representatives of subtypes A and G that are similar to the most recent common ancestor of the two clades. There, the CRF02 clade is a sibling lineage to contemporary subtype A and G sequences, branching nearest to, but outside of, the clade based on more current sequences (Fig. 4. Sibling of A in Regions I, III and sibling of G in Region II). The topologies of the trees also suggested that the current CRF02 has undergone multiple recombination events, and some genomic regions of the first generation of CRF02 sequences were replaced by more recent sequences (Fig. 4. CRF02 is a descendent lineage of A in Regions V, VI, and a descendent lineage of G in Region VII). To assess whether sibling and descendent phylogenetic classifications indicate older and more recent fragments, respectively, we analyzed the correlation between sampling time point and the height of taxa from its subtype most recent common ancestor (sMRCA). The largest subtype G fragment (Region II) was sampled in 1991-2002 (N = 39 taxa) and showed a correlation of R = 0.41 between sampling time and tip height from its sMRCA (P < 0.01, F-test, linear regression). Likewise, the largest subtype A1 fragment (Region VI) which was sampled in 1985-2003 (N = 102 taxa) had R = 0.50 (P < 0.01, F-test, linear regression). Note that the correlation coefficient (R) is not dependent on the molecular clock being a constant rate clock, only that branches get longer with time; the P value does however depend on a linear trend estimation. Thus, our phylogenetic assignments of "old" and "new" are supported by the correlation between sampling time and growth of tip height from the respective sMRCA. The alignment quality was fairly even in terms of gap counts and the genetic diversity followed expected gene patterns (Additional file 1, Fig S1).

In agreement with our results, such second generation recombinants have been noted by others to be common [44]. Of particular interest, a recent argument, based on an analysis of Region IV suggests that CRF02 is a pure subtype and is a parent of the contemporary G clade which is the recombinant. This is in contrast to the current HIV nomenclature which suggests that the G clade is the parent and CRF02 the recombinant [25]. To clarify the confusing but critical argument, we investigated all CRF02 and G sequences derived from the Los Alamos HIV sequence database. While our tree suggested that CRF02 was inside the G clade in Region IV, there was no bootstrap support for this classification. Importantly, besides Region IV, the rest of the genome fragments (both A1 and G) had better bootstrap support and clearly indicated that G is a subtype and CRF02 a recombinant (Fig 4). Furthermore, a RIP analysis attempting to resolve the origin of Region IV (and others) showed that CRF02 was closer to a G maximum likelihood-inferred ancestor (G.anc) than to a G consensus of contemporary sequences (G.con) (CRF02 to G.anc = 0.0178 substitutions/site, and CRF02 to G.con = 0.0218 substitutions/site) (Fig 4B). The likelihood was p < 10^-8 ^that G.anc and G.con were the same (2ΔlnL = 34.5, general-time-reversible model with 9 site rates), but there were only two positions that differed in Region IV and thus this result should be interpreted with caution. For instance, the underlying model parameters could change if new sequences were included in the inference, potentially changing the state probabilities and the site likelihoods. Nevertheless, for Region IV, at this point the difference between G.anc and G.con are significant, CRF02 was found overall closer to G.anc, and at the two positions G.anc and G.con differed CRF02 was identical to G.anc, all together suggesting a more ancient origin of CRF02 Region IV. Also note that the RIP analysis showed that Region IV has the least power to resolve the phylogenetic classification of the CRF02 genome, because this region has the smallest amount of divergence (Fig 4B). This also explains the poor bootstrap support in Region IV tree. Further, although the sequences are highly similar, the maximum likelihood estimates of ancestral sequences of clades A and G should reflect better the ancestral state of the clade, incorporating phylogenetic information from the full M group tree, while the consensus sequences derived from contemporary A and G isolates slightly favors contemporary forms. Thus, the RIP analysis further supported the tree results that some sections of the CRF02 genome may have involved old recombination events from a time when the clades were beginning to diverge, and that some other regions were more likely to have involved more recent subtype A and G sequences. To avoid potential problems with the uncertainty of breakpoint locations, we also phylogenetically analyzed smaller sub-regions of the larger regions (I', II', and VI') and found consistent results with the presented larger region analyses. In conclusion, taken all regions of the CRF02 genome into account, our analyses show that CRF02 is a recombinant of both ancient and more recent A and G parents.

### The Chinese BC-recombinant epidemic was formed locally with limited contacts with most other Asian countries

**Figure 5:**
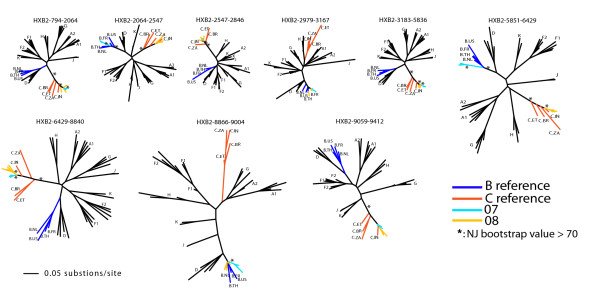
**ML trees of consensus sub-regions delimited by the jpHMM-derived breakpoints in CRF07 and CRF08 CRFs**. Bootstrap support values for clustering are shown.

**Figure 6:**
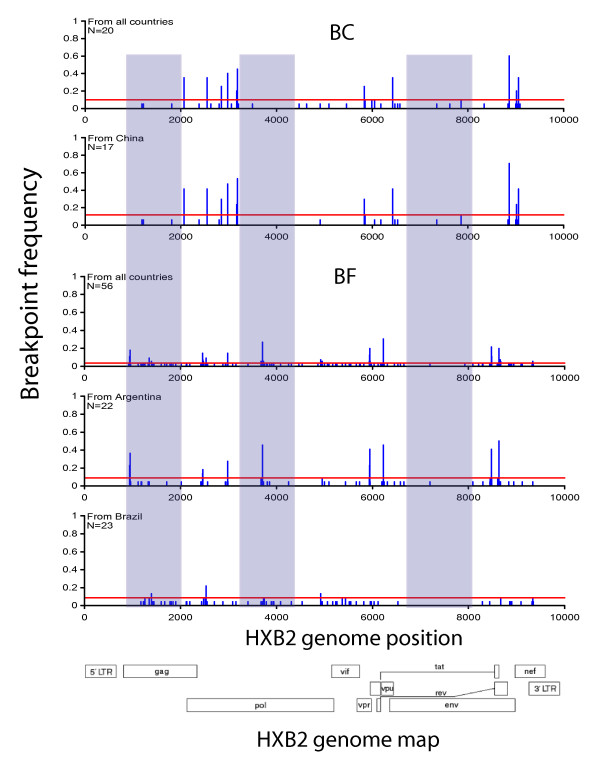
**Breakpoint frequency in near full-length BC and BF recombinants**. The breakpoint positions are based on the HXB2 numbering. Left and middle grey regions: genomic regions where breakpoints are less present in BC than BF recombinants. Right grey region: both BC and BF recombinants have few breakpoints within a segment of gp120. Vertical bars: the frequency of sequences with a breakpoint at that sequence position. Horizontal red lines: exactly 3 sequences sharing the breakpoint. Note that the frequency scales are different in each panel in order to maximize resolution.

To characterize the relationships of BC recombinants from China, Asia, and worldwide, we first investigated the relationship between CRF07 and CRF08. Full-length sequences classified as CRF07, CRF08, or BC were grouped according to their breakpoint structures (Fig. 2), and ML trees were constructed for sub-regions delimited by all CRF07 and CRF08 sequences (Fig. 5). While most of the examined sub-regions showed a sibling relationship between CRF07 and CRF08, two sub-regions (HXB2 positions 794-2064 and 2547-2846) suggested that, at least in these sub-regions, CRF08 may be the parent of CRF07 because CRF07 sequences were clustered inside the CRF08 clade (bootstrap support ≥ 70%). Further, CRF07 and CRF08 were derived from multiple recombination events, as indicated by unequal breakpoint frequencies in CRF07 and CRF08 (Fig. 6, top panels). The breakpoint at HXB2 position 8866 was consistent among CRF07, CRF08, and subsequent recombinants, and thus was likely to be introduced into CRF07 and CRF08 through a common ancestor.

To investigate BC recombinants from China and China's neighboring countries, phylogenetic analyses were performed on consensus sub-regions delimited by most near-full-length BC recombinants shown in Figure 2. There was a close relationship between Yunnan B and Myanmar B (data not shown). Sequences from these two geographic regions are very limited (6 BC sequences from Yunnan and 2 from Myanmar), therefore we cannot deduce the direction of the epidemic movement between Yunnan and Myanmar.

Finally, the influence of worldwide B and C epidemics on the Chinese BC recombinants was analyzed. As described in the Materials and Methods, the global set of subtype B and C sequences was retrieved from the HIV database in the genomic regions that had the longest subtype B and the longest C sub-regions shared by all CRF07, CRF08, and most near full-length BC recombinants. In the subtype B sub-region tree, sequences from China appeared to be a local epidemic only involving neighboring countries Thailand and Myanmar (Additional file 1 Fig. S2A); this occurred possibly through drug trafficking routes [26,28]. Other Asian countries, for instance, Korea, Japan, and Thailand, appeared to have greater subtype B diversity, which may be explained by more frequent contacts with each other and with the rest of the world. Finally, South American subtype B seems to have had multiple HIV introductions from Europe and North America. The result of the subtype C sub-region tree also suggested that China C is a mostly local epidemic, with some influx of subtype C from India, but not Africa as India has (Additional file 1, Fig. S2B). Finally, the dominant South American C epidemic appears to have derived from a single introduction from Africa ([45,46] and Additional file 1, Fig. S2).

### Contemporary Argentinean and Brazilian HIV epidemics are not independent

Our study did not show any association between risk factors and BF CRF groups (Fig. 3). In the breakpoint frequency analyses of full-length BF sequences (Figure 6, BF panels) and BF sequence fragments (Additional file 1, Fig. S3) all identified BF breakpoints were found in more than one country in South America, and occasionally in countries from other continents. This suggests that, although the South American HIV epidemic is represented by two distinctive epicenters, the BF epidemic has moved back and forth between Argentina and Brazil. Indeed, the BF recombinant sequence fragments carry all the information that fills the gap in the full-genome sequences from Argentina and Brazil such that all genomic regions of B and F can be found in either country. We also found that sequence V62 (accession number AY536236), which has an epidemiological linkage to the Argentinean epidemic [47], had the same genomic structure and breakpoints as CRF28, which was first described in Brazil. In all, the HIV epidemics in Argentina and Brazil are not independent.

We did not find evidence that Argentinean B and F were derived from Brazil, as previously suggested [47,48]. The result of the phylogenetic analyses, which agreed with previous publications [40,49,50] and thus not shown here, demonstrated that B and F fragments of the jpHMM-confirmed CRF12, CRF28, and CRF29 were inter-mingled, and therefore could not support a single direction of HIV-1 flow. Also, as already mentioned, we found that Argentinean B and F sequence fragments in the HIV database can cover a full HIV-1 genome of each subtype, meaning that there was a potential to form any BF recombinants in Argentina and that there was no need to assume that already-recombined genomes came from Brazil. In addition, a recently identified near full-length Argentinean pure F sequence, ARE933 (accession number DQ189088), was found to be closer to Argentinean BF than were any other F strains [41,51]. The most likely scenario is that there were HIV-1 transmissions in both directions, with recombination of circulating strains in all countries involved.

## Discussion

The geographic distribution of subtypes and recombinant lineages in any epidemic, influenced by local epidemiological factors, is dynamic and difficult to resolve. Here we present a large-scale subtyping re-analysis of 9435 HIV-1 sequences that involve subtypes A, B, C, G, F, and their important CRFs in three different epidemiological settings that together have significantly shaped today's global HIV epidemic. Our comprehensive analyses demonstrate strong evidence that the contemporary HIV-1 epidemic is a mixture of recombinants that had an origin in the early HIV epidemic, likely before the subtypes were distinctively separated, while others are of more recent origin, and that shared breakpoints can be used for tracking patterns in the epidemic.

We found that CRF02 is a recombinant more complex than previously described. Its old origin, as well as the subsequent recombination events that occurred prior to the establishment of the contemporary CRF02 lineage, can easily confound the analysis of CRF02. Among the BC recombinants we found that the BC epidemic in China is unique compared to most other Asian countries; further, CRF07 and CRF08 were recently introduced to the epidemic, but both have undergone multiple recombination events. The study of BF recombinants in South Africa suggests that the HIV-1 epidemics in Argentina and Brazil are not independent.

**Figure 7:**
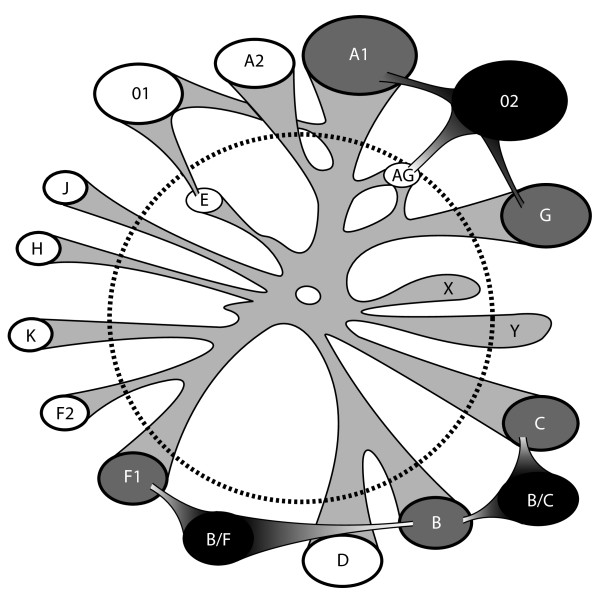
**The current HIV-1 epidemic is a mixture of old and contemporary lineages**. The dashed circle differentiates old and contemporary sequences. Inside the circle, the old sequences, such as the subtype E clade, may no longer exist in the current epidemic. We can only infer the ancient presence of subtype E based on CRF01_AE, a recombinant between subtype A and E. "X" represents a hypothetical extinct strain, "Y" represents a hypothetical old strain that is still circulating in the current epidemic, but hasn't been identified. CRF02 is an old recombinant derived from both old and contemporary subtype A and G. BF recombinants in South America and BC in China are new, as their parents are contemporary sequences. The black blobs (recombinants) and grey blobs ("pure" subtypes) are clades investigated in this paper, and white blobs are other HIV-1 clades.

The existence of early lineages in the current HIV-1 epidemic imposes a great challenge in detecting some recombinant sequences. Figure 7 shows a cartoon describing some of the difficulties described in this paper (e.g. CRF02) and also some effects of extinct (e.g. subtype "E") and undiscovered lineages. In addition to recombination effects, co-evolution of some sequence positions, for example due to fitness constrains and HLA-imposed immune pressure, gives rise to distinct but potentially convergent patterns of immune escape that can also confound recombination analyses by introducing homoplasy. Sometimes the history of old lineages can be recovered by extrapolating backward from surviving viruses (like subtype E [52,53]), while some lineages presumably can never be found (like lineage "X" in Fig. 7). In this context, it is likely that [some of] the current pure subtypes are actually recombinants that were formed a long time ago, but because the "pure" parental lineages have been lost, we cannot trace their origin. Thus the current subtype nomenclature does not rest on the assumption that currently defined "pure" subtypes are not consequences of earlier recombination events, but rather indicates that these subtypes can be used as good background references in studying the current HIV-1 epidemic, and that the "pure" subtypes' relative genetic relatedness can provide a basis for studying and understanding the immunological consequences of diversity for vaccine design. Unfortunately, almost all existing genotyping tools are not well designed to infer old recombination events or for those that involve unknown parents.

The dynamic HIV-1 epidemic seems to have moved toward to have more complex recombinants. However the driving force may be different in different epidemiological settings. In Africa where the HIV epidemic is predominantly driven by heterosexual transmissions, the ancient history of CRF02 as described in this paper, together with its high replicative capacity [54,55] and its high prevalence [56], make CRF02 an active participant in generating more and new complex recombinants, for instance, the newly identified CRF36_cpx [57]. BC recombinants in China will likely also continue to evolve. Super-infection of CRF07 and CRF08 viruses [28], as well as continuous influx of B and C into Yunnan from China's surrounding countries [58,59], contributes greatly to the emergence of new BC recombinants, notably BC URFs. Another important driving force of BC evolution in China is the rapid transition in the HIV-1 epidemic in some geographical regions. In Yunnan alone, subtype B was found to be the dominant subtype in the late 1980s, but it was soon replaced by Thai B; in 1992, subtype C was found in this region, thus Thai B and C co-circulated; in 1994, CRF01 was identified in Yunnan; in 2000 and 2001, subtype C was not detected among IDU samples in the same region [28,30,37,58]. While some of these transitions in the regional prevalence might have been a consequence of sampling biases, they still suggest complex patterns of epidemic dynamics. BF recombinants in South America are possibly moving toward having more URFs. A recent Bayesian hierarchical analysis also indicated extensive ongoing recombination among CRF12 viruses [60]. The long circulation record of subtypes B and F in South America [43,61,62], and the tight HIV-1 transmission networks with high incidence rates found in some South American geographical regions may favor an elevated number of dual- and super-infections [48]. A possible outcome of this dynamic pattern is that pure subtype F may disappear after being gradually diluted from the South American epidemic. Hence, social network structures and possibly viral factors dictate the molecular epidemiology of HIV-1 [63]; and tracking the genetic lineages and patterns in recombination breakpoints can shed light on such factors.

Current CRF nomenclature requires all sequences of one CRF to have identical or very similar breakpoints, and thus originate from a single lineage of a recombinant form. Such breakpoints may, however, be easily blurred by subsequent substitutions and by further recombination events, as in the CRF02 and CRF17 cases described above. Hence, the sequences defined in a CRF are merely snapshots of the dynamic changes in HIV-1 evolution. This may cause problems when "new" CRFs are identified, which may be related to existing CRFs and not fit into meaningful groupings used for molecular epidemiology or vaccine design. It is questionable if the continuous use of these soon to be unmanageable number of CRFs will be useful to the HIV research community. Hence, we propose to define sequence "families" that would contain recombinant sequences composed of the same subtypes, but the sequences' genomic structures and breakpoints may not be identical due to successive recombination events or our inability to accurately describe them. Sequences would belong to one family as long as they are closer to a defined central strain of that family than to any other family, including "pure" subtypes, like the examples shown in Figs. 1, 2, 3. Each family would be defined by a central sequetype and the radius of family members would depend on distances in a multi-dimensional sequence space. Membership to a family could be based on overall similarity and, if needed, be further assessed by multidimensional scaling. Using such a "family" concept makes it feasible to dynamically track HIV diversity and epidemiologically important families over evolutionary time, regardless of their precise phylogenetic history.

## Conclusions

Our large-scale re-subtyping meta-study provides a comprehensive view of HIV recombinants in three epidemically important regions. The dynamic HIV epidemic is moving toward having increasing complexity and higher prevalence of recombinant forms and it has posed a great challenge in many aspects of the HIV/AIDS epidemic. We suggest that a revision of some CRFs may be needed. As we continue to systematically re-subtype the rest of the sequences in the Los Alamos sequence database, it is likely that our results will shed light on the impact of evolving HIV recombinants. This will give better database search results and may thus affect vaccine design and molecular epidemiology studies.

## Methods

### Sequences

The following sequence sets were retrieved from the Los Alamos HIV sequence database (http://www.hiv.lanl.gov sequence database search interface). Set 1: All near full-length sequences (>7000 nucleotides [nt]) of subtypes A, B, C, F, G, CRF02, CRF07, CRF08, CRF12, CRF17, CRF28, CRF29, and all URFs composed exclusively of subtypes A and G, or B and C, or B and F. Set 2: Shorter HIV sequences (300 - 7000 nt) that are BC recombinants from Asia and BF recombinants from South America. Set 3: After examining all full-length BC recombinant genomes, we defined the longest subtype B segment (HXB2 positions 3497-4473) and the longest subtype C region (HXB2 positions 6582-7349). For additional BC analyses, sequences covering these two fragments were retrieved from the database for all geographic regions worldwide. To avoid redundancy and reduce issues related to non-independence of data points, only 1 sequence per patient was included in the analyses of the sequence fragments. The analysis method used for Set 3 sequences, however, is not applicable to the AG and BF sequences due to the difficulties in obtaining accurate breakpoints (which is the case of CRF02; its old origin leads to fuzzy breakpoints) and in getting big enough genome regions (this is the case of South American BF in which URFs outnumber CRFs).

To examine further whether the risk factors contribute to the spread of BF URFs in South America, the associated risk factor of the near full-length BF recombinant sequences from South America was also retrieved from the database.

All sequences were aligned with HIV-1 subtype reference sequences (http://www.hiv.lanl.gov sequence alignment page) using GeneCutter (http://www.hiv.lanl.gov GeneCutter page). Alignment quality was checked manually in BioEdit [64] to ensure that the alignments did not contain obvious problems and that they were correctly codon aligned.

### Recombination detection

Our jumping profile hidden Markov model (jpHMM) program [65,66] was used to analyze the subtype assignment of all sequences retrieved. In jpHMM, each HIV-1 subtype is represented by a profile hidden Markov model. All profile models are connected by empirical probabilities, allowing the detection of possible recombinants and related breakpoints by jumping from one profile to another. jpHMM performs best in predicting recombinants that involve subtypes that have had adequate sampling to build well-informed profiles, i.e., it is less effective for subtypes H, J, and K, because so few full-length genome sequences are available (N = 3, 3, and 2, respectively). In the present study, jpHMM was used to detect the recombination patterns in recombinants that are composed exclusively of subtypes A and G, B and C, or B and F; each of these subtypes has enough data to form a good model of sequence variation. For the recombinants detected, jpHMM provides detailed information about subtype composition and well-resolved breakpoint locations. The jpHMM source code is available at the jpHMM Web interface http://jphmm.gobics.de.

### Phylogenetic analyses

The near full-length sequences were grouped together if the sequences had similar subtype composition and breakpoint patterns. Sub-genomic regions delimited by shared breakpoints in the majority of AG recombinants (including jpHMM-confirmed CRF02 and AG URFs) were further analyzed using phylogenetic inference, discriminating between parental, descendent and sibling relationships. Initial screens of regions involving thousands of taxa, for example the global collection of the largest identified B and C genomic sub-regions in all near full-length BC recombinants (Set 3), were performed using PAUP neighbor joining with an F84 model [67]. More refined resolution of B and C recombinants, and all other primary trees involving sub-regions delimited by shared jpHMM-confirmed breakpoints, were done using PhyML with a GTR-Gamma model, enabling very large datasets to be analyzed phylogenetically [68]. The statistical robustness and the reliability of the notable clustering patterns in the ML trees were further evaluated by non-parametric bootstrap analyses in PAUP (neighbor-joining, F84 model, 1000 replicates). A bootstrap value of ≥ 70% was considered significant for subtype clustering [69].

### Further analysis of CRF02 origin

The Recombination Identification Program version 3 (http://www.hiv.lanl.gov RIP3 page) was used along with likelihood trees to examine the relationship between CRF02 and contemporary sequences relative to maximum likelihood inferred ancestor sequences of subtypes A and G: A CRF02 consensus sequence was analyzed against an alignment that included ML-inferred ancestral sequences [70] (M group, A1, and G) and consensus sequences (M group, A1, and G). The CRF02 consensus was constructed based on the CRF02 sequences sets confirmed by jpHMM and phylogenetic analysis. Other consensus and ancestral sequences were retrieved from the HIV sequence database alignment page. All consensus and ancestral sequences were aligned using GeneCutter, followed by manual editing.

### Breakpoint frequency calculations

Systematic breakpoint frequency calculations were performed on the following three sequence alignments: near full-length BC and BF recombinant sequences; fragments of BC recombinant sequences from Asia; and fragments of BF recombinant sequences from South America. The sequence subtyping and recombination patterns were derived from jpHMM analyses. The breakpoint frequencies of all sequences in each alignment were calculated and plotted. In BC CRFs, > 95% of the breakpoints were located within 16 nt from the breakpoint median. In BF CRFs, > 95% of the breakpoints were located within 98 nt from the breakpoint median. These two numbers (16 nt and 98 nt) were used as breakpoint confidence regions for subsequent analyses of BC and BF recombinants, respectively, to provide boundaries for defining shared breakpoints.

## Competing interests

The authors declare that they have no competing interests.

## Authors' contributions

TL, MZ and BK designed and carried out the genotyping procedure. MZ, TL, BK, BF and JM analyzed the data. AS, MZ, BM, TL, IB, MS, BK developed and evaluated the jpHMM software. MZ, TL and BK wrote the manuscript. All authors approved the final version of the manuscript.

## Supplementary Material

Additional file 1 **Supplementary figures S1, S2, S3**. Suppl. figure 1. Gap frequency and mean pairwise distance in the CRF02 alignment. Suppl. figure 2. The BC epidemic in China is unique compared to China's neighboring countries. Suppl. figure 3. The breakpoint frequency of CRF12, CRF28, and CRF29 sequences. Click here for file
