# Supplementary material for: The role of recombination in the emergence of a complex and dynamic HIV epidemic
Source: Retrovirology. 2010 Mar 23;7:25. doi: 10.1186/1742-4690-7-25 (PMC2855530; doi:10.1186/1742-4690-7-25)
Supplement: Additional file 1 — Supplementary figures S1, S2, S3. Suppl. figure 1. Gap frequency and mean pairwise distance in the CRF02 alignment. Suppl. figure 2. The BC epidemic in China is unique compared to China's neighboring countries. Suppl. figure 3. The breakpoint frequency of CRF12, CRF28, and CRF29 sequences. [file 1742-4690-7-25-S1.PDF]

THE ROLE OF RECOMBINATION IN THE EMERGENCE OF A COMPLEX AND DYNAMIC HIV EPIDEMIC

Ming Zhang, Brian Foley, Anne-Kathrin Schultz, Jennifer P. Macke, Ingo Bulla, Mario Stanke, Burkhard Morgenstern, Bette Korber, Thomas Leitner

Supplementary figures:

Suppl. figure 1. Plot showing that our CRF02 conclusion, that is, CRF02 was derived from old and new recombination events, is not biased by the CRF02 sequence alignment quality.

Gap%: percentage of alignment gaps within every 500 nt window.

Mean dist: mean evolutionary distance (F84) within every 500 nt window.

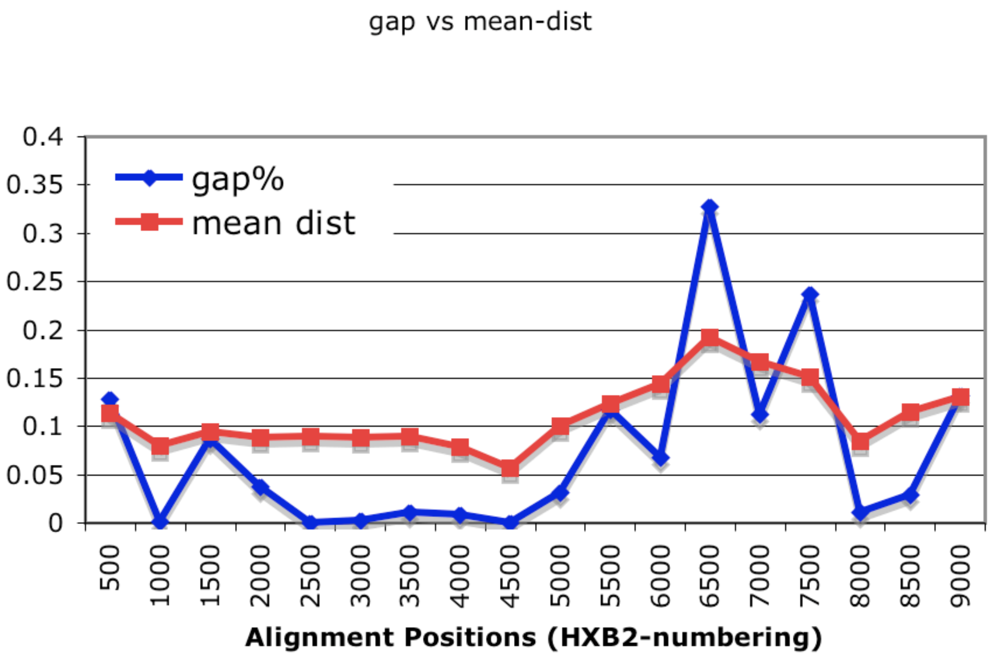

Suppl. figure 2. The BC epidemic in China is unique compared to China's neighboring countries. Two neighbor-joining trees showing that China B and C are more restrictedly derived from China's neighboring countries, while the B and C sequences from China neighboring countries have more contacts with B and C from other regions of the world. Sequences used here were obtained from the worldwide sources (Set 3, as described in Methods). Each sequence name is in a standard format of number.subtype.country, where the number is a sequential identifier used in the sequence alignments.

(A) Subtype B sequence fragments (HXB2 positions: 3497-4473)

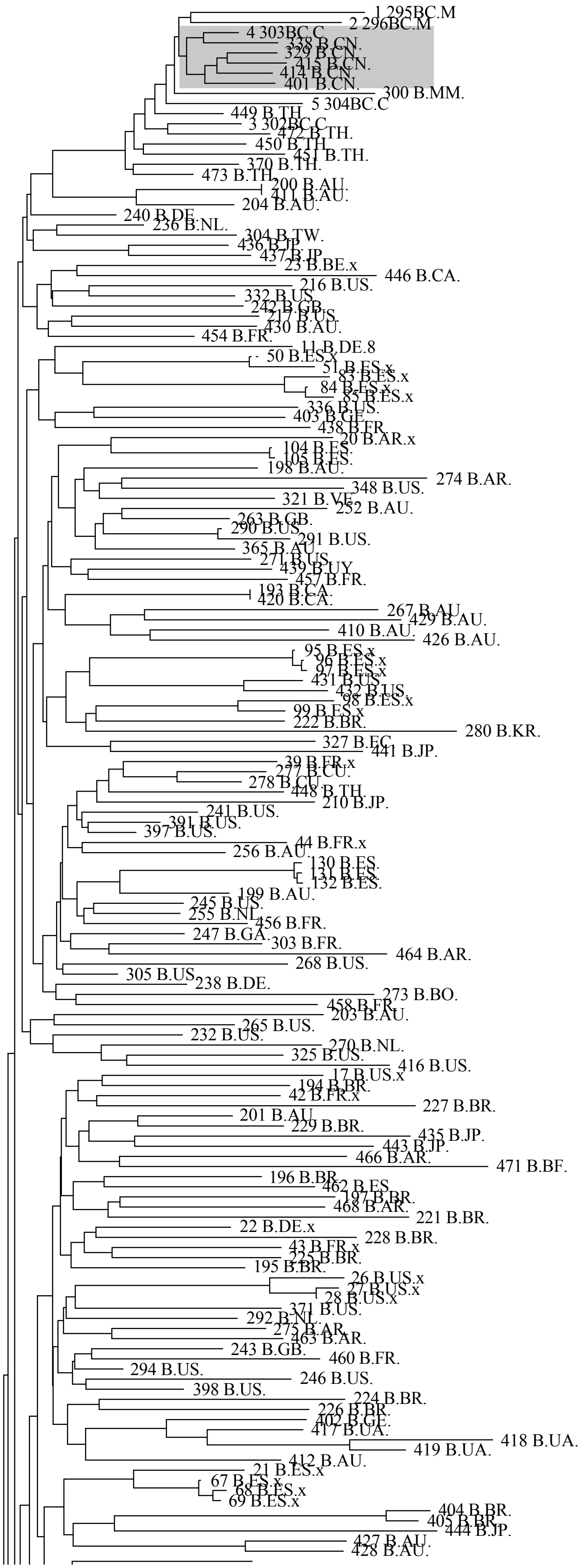

1 295BC.M
2 296BC.M
4 303BC.C
401 B.CN.
300 B.MM.
5 304BC.C
449 B.TH.
3 302BC.C
472 B.TH.
450 B.TH.
451 B.TH.
370 B.TH.
473 B.TH.
200 B.AU.
411 B.AU.
204 B.AU.
240 B.DE.
236 B.NL.
304 B.TW.
436 B.JP.
437 B.JP.
23 B.BE.x
446 B.CA.
216 B.US.
332 B.US.
242 B.GB.
217 B.US.
430 B.AU.
454 B.FR.
11 B.DE.8
50 B.ES.x
51 B.ES.x
83 B.ES.x
84 B.ES.x
85 B.ES.x
336 B.US.
403 B.GE.
438 B.FR.
20 B.AR.x
104 B.ES.
105 B.ES.
198 B.AU.
274 B.AR.
348 B.US.
321 B.VE.
252 B.AU.
263 B.GB.
290 B.US.
291 B.US.
365 B.AU.
271 B.US.
439 B.UY.
457 B.FR.
193 B.CA.
420 B.CA.
267 B.AU.
429 B.AU.
410 B.AU.
426 B.AU.
95 B.ES.x
96 B.ES.x
97 B.ES.x
431 B.US.
432 B.US.
98 B.ES.x
99 B.ES.x
222 B.BR.
280 B.KR.
327 B.EC.
441 B.JP.
39 B.FR.x
277 B.CU.
278 B.CU.
448 B.TH.
210 B.JP.
241 B.US.
391 B.US.
397 B.US.
44 B.FR.x
256 B.AU.
130 B.ES.
131 B.ES.
132 B.ES.
199 B.AU.
245 B.US.
255 B.NL.
456 B.FR.
247 B.GA.
303 B.FR.
464 B.AR.
268 B.US.
305 B.US.
238 B.DE.
273 B.BO.
458 B.FR.
203 B.AU.
265 B.US.
232 B.US.
270 B.NL.
325 B.US.
416 B.US.
17 B.US.x
194 B.BR.
42 B.FR.x
227 B.BR.
201 B.AU.
229 B.BR.
435 B.JP.
443 B.JP.
466 B.AR.
471 B.BF.
196 B.BR.
462 B.ES.
197 B.BR.
468 B.AR.
221 B.BR.
22 B.DE.x
228 B.BR.
43 B.FR.x
225 B.BR.
195 B.BR.
26 B.US.x
27 B.US.x
28 B.US.x
371 B.US.
292 B.NL.
275 B.AR.
463 B.AR.
243 B.GB.
460 B.FR.
294 B.US.
246 B.US.
398 B.US.
224 B.BR.
226 B.BR.
402 B.GE.
417 B.UA.
418 B.UA.
419 B.UA.
412 B.AU.
21 B.ES.x
67 B.ES.x
68 B.ES.x
69 B.ES.x
404 B.BR.
405 B.BR.
444 B.JP.
427 B.AU.
428 B.AU.

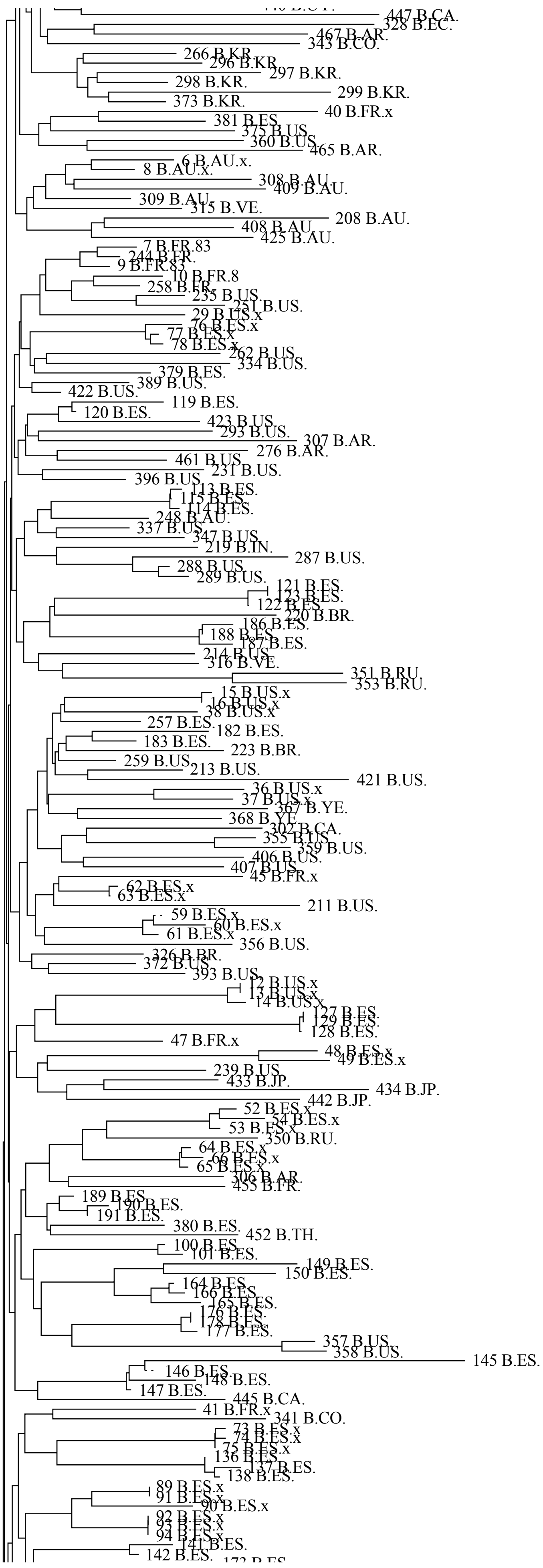
447 B.CA.
328 B.EC.
467 B.AR.
343 B.CO.
266 B.KR.
296 B.KR.
297 B.KR.
298 B.KR.
299 B.KR.
373 B.KR.
40 B.FR.x
381 B.ES.
375 B.US.
360 B.US.
465 B.AR.
6 B.AU.x.
8 B.AU.x.
308 B.AU.
409 B.AU.
309 B.AU.
315 B.VE.
208 B.AU.
408 B.AU.
425 B.AU.
7 B.FR.83
244 B.FR.
9 B.FR.83
10 B.FR.8
258 B.FR.
235 B.US.
251 B.US.
29 B.US.x
76 B.ES.x
77 B.ES.x
78 B.ES.x
262 B.US.
334 B.US.
379 B.ES.
389 B.US.
422 B.US.
119 B.ES.
120 B.ES.
423 B.US.
293 B.US.
307 B.AR.
276 B.AR.
461 B.US.
231 B.US.
396 B.US.
113 B.ES.
115 B.ES.
114 B.ES.
248 B.AU.
337 B.US.
347 B.US.
219 B.IN.
287 B.US.
288 B.US.
289 B.US.
121 B.ES.
123 B.ES.
122 B.ES.
220 B.BR.
186 B.ES.
188 B.ES.
187 B.ES.
214 B.US.
316 B.VE.
351 B.RU.
353 B.RU.
15 B.US.x
16 B.US.x
38 B.US.x
257 B.ES.
182 B.ES.
183 B.ES.
223 B.BR.
259 B.US.
213 B.US.
421 B.US.
36 B.US.x
37 B.US.x
367 B.YE.
368 B.YE.
302 B.CA.
355 B.US.
359 B.US.
406 B.US.
407 B.US.
45 B.FR.x
62 B.ES.x
63 B.ES.x
211 B.US.
59 B.ES.x
60 B.ES.x
61 B.ES.x
356 B.US.
326 B.BR.
372 B.US.
393 B.US.
12 B.US.x
13 B.US.x
14 B.US.x
127 B.ES.
129 B.ES.
128 B.ES.
47 B.FR.x
48 B.ES.x
49 B.ES.x
239 B.US.
433 B.JP.
434 B.JP.
442 B.JP.
52 B.ES.x
54 B.ES.x
53 B.ES.x
350 B.RU.
64 B.ES.x
66 B.ES.x
65 B.ES.x
306 B.AR.
455 B.FR.
189 B.ES.
190 B.ES.
191 B.ES.
380 B.ES.
452 B.TH.
100 B.ES.
101 B.ES.
149 B.ES.
150 B.ES.
164 B.ES.
166 B.ES.
165 B.ES.
176 B.ES.
178 B.ES.
177 B.ES.
357 B.US.
358 B.US.
145 B.ES.
146 B.ES.
148 B.ES.
147 B.ES.
445 B.CA.
41 B.FR.x
341 B.CO.
73 B.ES.x
74 B.ES.x
75 B.ES.x
136 B.ES.
137 B.ES.
138 B.ES.
89 B.ES.x
91 B.ES.x
90 B.ES.x
92 B.ES.x
93 B.ES.x
94 B.ES.x
141 B.ES.
142 B.ES.

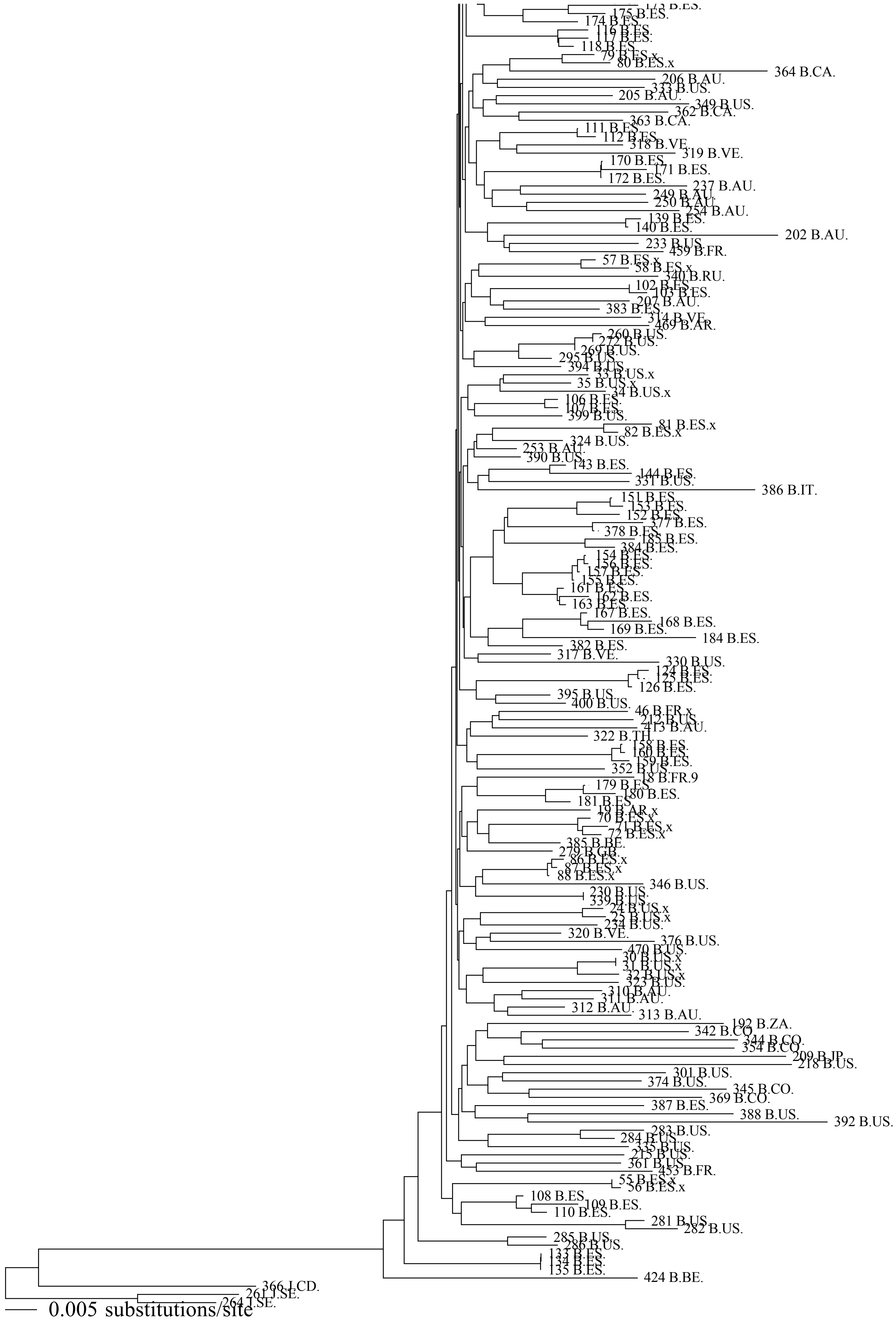
175 B.ES.
174 B.ES.
118 B.ES.
79 B.ES.x
80 B.ES.x
364 B.CA.
206 B.AU.
333 B.US.
205 B.AU.
349 B.US.
362 B.CA.
363 B.CA.
111 B.ES.
112 B.ES.
318 B.VE.
319 B.VE.
170 B.ES.
171 B.ES.
172 B.ES.
237 B.AU.
249 B.AU.
250 B.AU.
254 B.AU.
139 B.ES.
140 B.ES.
202 B.AU.
233 B.US.
459 B.FR.
57 B.ES.x
58 B.ES.x
340 B.RU.
102 B.ES.
103 B.ES.
207 B.AU.
383 B.ES.
314 B.VE.
469 B.AR.
260 B.US.
272 B.US.
269 B.US.
295 B.US.
394 B.US.
33 B.US.x
35 B.US.x
34 B.US.x
106 B.ES.
399 B.US.
81 B.ES.x
82 B.ES.x
324 B.US.
253 B.AU.
390 B.US.
143 B.ES.
144 B.ES.
331 B.US.
386 B.IT.
151 B.ES.
153 B.ES.
152 B.ES.
377 B.ES.
378 B.ES.
185 B.ES.
384 B.ES.
154 B.ES.
156 B.ES.
157 B.ES.
155 B.ES.
161 B.ES.
162 B.ES.
163 B.ES.
167 B.ES.
168 B.ES.
169 B.ES.
184 B.ES.
382 B.ES.
317 B.VE.
330 B.US.
124 B.ES.
125 B.ES.
126 B.ES.
395 B.US.
400 B.US.
46 B.FR.x
212 B.US.
413 B.AU.
322 B.TH.
158 B.ES.
160 B.ES.
159 B.ES.
352 B.US.
18 B.FR.9
179 B.ES.
180 B.ES.
181 B.ES.
19 B.AR.x
70 B.ES.x
71 B.ES.x
72 B.ES.x
385 B.BE.
279 B.GB.
86 B.ES.x
87 B.ES.x
88 B.ES.x
346 B.US.
230 B.US.
339 B.US.
24 B.US.x
25 B.US.x
234 B.US.
320 B.VE.
376 B.US.
470 B.US.
30 B.US.x
31 B.US.x
32 B.US.x
323 B.US.
310 B.AU.
311 B.AU.
312 B.AU.
313 B.AU.
192 B.ZA.
342 B.CO.
344 B.CO.
354 B.CO.
209 B.JP.
218 B.US.
301 B.US.
374 B.US.
345 B.CO.
369 B.CO.
387 B.ES.
388 B.US.
392 B.US.
283 B.US.
284 B.US.
335 B.US.
215 B.US.
361 B.US.
453 B.FR.
55 B.ES.x
56 B.ES.x
108 B.ES.
109 B.ES.
110 B.ES.
281 B.US.
282 B.US.
285 B.US.
286 B.US.
135 B.ES.
424 B.BE.
366 J.CD.
261 J.SE.
264 J.SE.
0.005 substitutions/site

(B) Subtype C sequence fragments: HXB2 positions: 6582-7349.

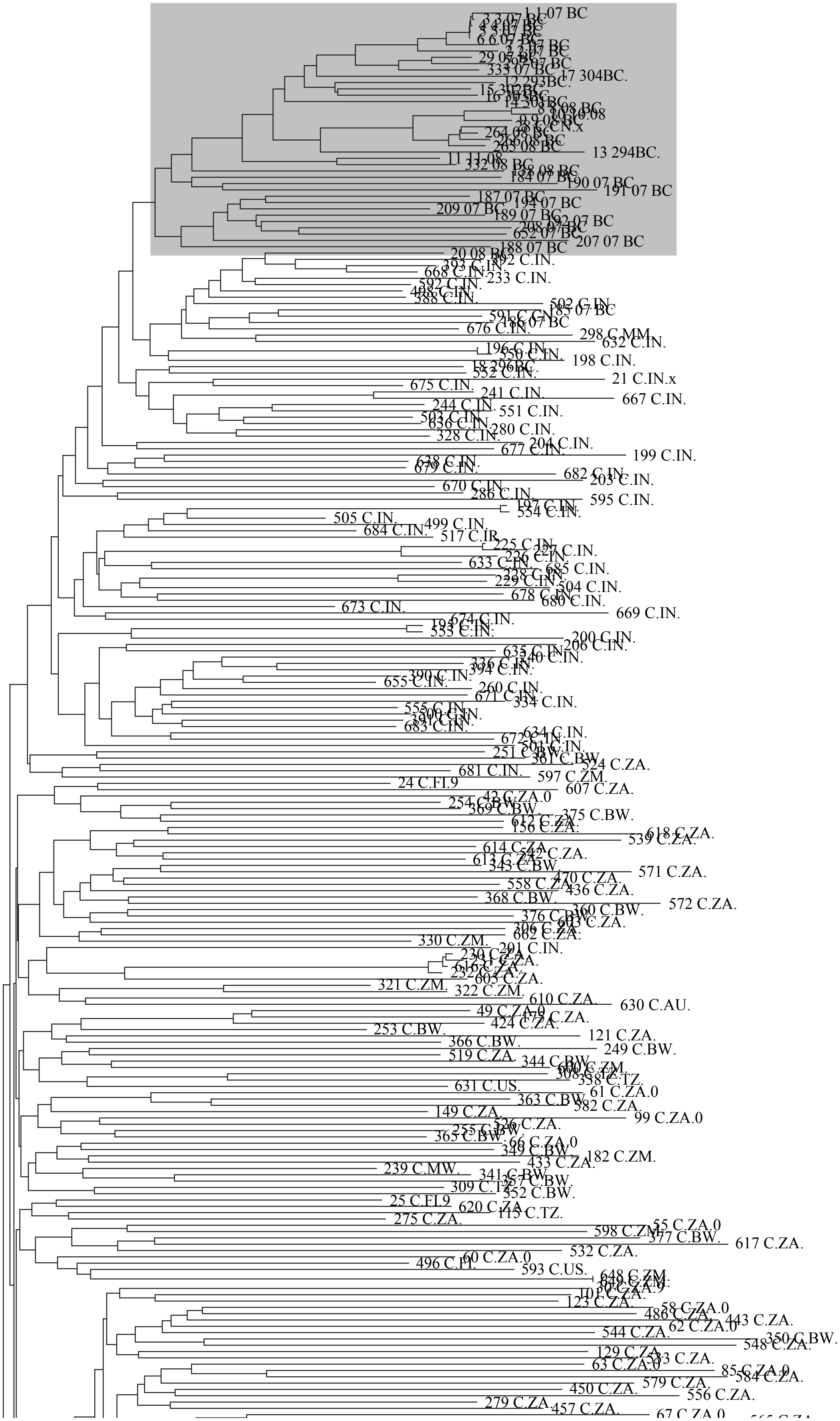
1 1 07 BC
17 304BC.
13 294BC.
190 07 BC
191 07 BC
194 07 BC
209 07 BC
207 07 BC
188 07 BC
233 C.IN.
388 C.IN.
185 07 BC
676 C.IN.
632 C.IN.
198 C.IN.
21 C.IN.x
675 C.IN.
241 C.IN.
667 C.IN.
551 C.IN.
280 C.IN.
328 C.IN.
204 C.IN.
677 C.IN.
199 C.IN.
679 C.IN.
203 C.IN.
595 C.IN.
554 C.IN.
505 C.IN.
499 C.IN.
684 C.IN.
517 C.IR.
227 C.IN.
685 C.IN.
504 C.IN.
680 C.IN.
673 C.IN.
669 C.IN.
674 C.IN.
553 C.IN.
200 C.IN.
206 C.IN.
640 C.IN.
394 C.IN.
655 C.IN.
260 C.IN.
334 C.IN.
683 C.IN.
634 C.IN.
501 C.IN.
361 C.BW.
324 C.ZA.
681 C.IN.
597 C.ZM.
24 C.FI.9
607 C.ZA.
42 C.ZA.0
369 C.BW.
375 C.BW.
156 C.ZA.
618 C.ZA.
539 C.ZA.
242 C.ZA.
343 C.BW.
571 C.ZA.
470 C.ZA.
558 C.ZA.
436 C.ZA.
368 C.BW.
572 C.ZA.
360 C.BW.
376 C.BW.
662 C.ZA.
330 C.ZM.
201 C.IN.
603 C.ZA.
321 C.ZM.
322 C.ZM.
610 C.ZA.
630 C.AU.
49 C.ZA.0
424 C.ZA.
253 C.BW.
121 C.ZA.
366 C.BW.
249 C.BW.
519 C.ZA.
344 C.BW.
338 C.TZ.
631 C.US.
61 C.ZA.0
363 C.BW.
582 C.ZA.
149 C.ZA.
99 C.ZA.0
526 C.ZA.
365 C.BW.
66 C.ZA.0
349 C.BW.
182 C.ZM.
433 C.ZA.
239 C.MW.
341 C.BW.
352 C.BW.
25 C.FI.9
620 C.ZA.
115 C.TZ.
275 C.ZA.
55 C.ZA.0
598 C.ZM.
377 C.BW.
617 C.ZA.
532 C.ZA.
60 C.ZA.0
496 C.PT.
593 C.US.
648 C.ZM.
123 C.ZA.
58 C.ZA.0
486 C.ZA.
443 C.ZA.
544 C.ZA.
62 C.ZA.0
350 C.BW.
548 C.ZA.
129 C.ZA.
583 C.ZA.
63 C.ZA.0
85 C.ZA.0
584 C.ZA.
450 C.ZA.
579 C.ZA.
556 C.ZA.
279 C.ZA.
457 C.ZA.
67 C.ZA.0

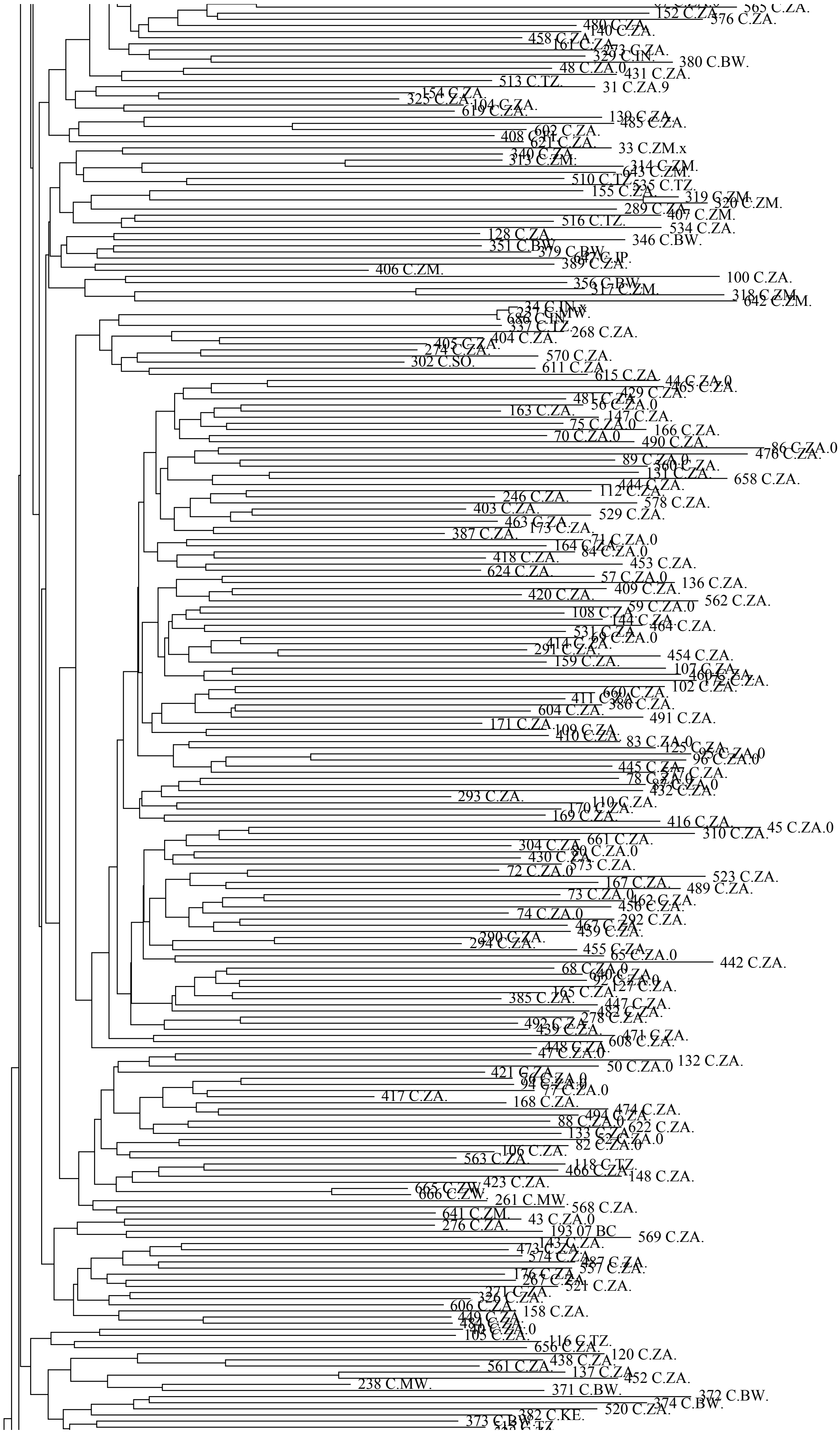
565 C.ZA.
152 C.ZA.
376 C.ZA.
140 C.ZA.
458 C.ZA.
329 C.IN.
380 C.BW.
48 C.ZA.0
431 C.ZA.
513 C.TZ.
31 C.ZA.9
154 C.ZA.
104 C.ZA.
619 C.ZA.
485 C.ZA.
602 C.ZA.
408 C.ZA.
621 C.ZA.
33 C.ZM.x
315 C.ZM.
314 C.ZM.
510 C.TZ.
535 C.TZ.
155 C.ZA.
319 C.ZM.
320 C.ZM.
289 C.ZA.
407 C.ZM.
516 C.TZ.
534 C.ZA.
128 C.ZA.
346 C.BW.
351 C.BW.
379 C.BW.
389 C.ZA.
406 C.ZM.
100 C.ZA.
356 C.BW.
517 C.ZM.
318 C.ZM.
642 C.ZM.
686 C.IN.
337 C.TZ.
268 C.ZA.
404 C.ZA.
405 C.ZA.
274 C.ZA.
570 C.ZA.
302 C.SO.
611 C.ZA.
615 C.ZA.
44 C.ZA.0
465 C.ZA.
429 C.ZA.
481 C.ZA.
56 C.ZA.0
163 C.ZA.
147 C.ZA.
75 C.ZA.0
166 C.ZA.
70 C.ZA.0
490 C.ZA.
86 C.ZA.0
476 C.ZA.
89 C.ZA.0
360 C.ZA.
131 C.ZA.
658 C.ZA.
444 C.ZA.
246 C.ZA.
112 C.ZA.
578 C.ZA.
403 C.ZA.
529 C.ZA.
463 C.ZA.
173 C.ZA.
387 C.ZA.
71 C.ZA.0
164 C.ZA.
84 C.ZA.0
418 C.ZA.
453 C.ZA.
624 C.ZA.
57 C.ZA.0
136 C.ZA.
420 C.ZA.
409 C.ZA.
562 C.ZA.
108 C.ZA.
59 C.ZA.0
144 C.ZA.
464 C.ZA.
531 C.ZA.
414 C.ZA.
291 C.ZA.
454 C.ZA.
159 C.ZA.
107 C.ZA.
102 C.ZA.
660 C.ZA.
411 C.ZA.
386 C.ZA.
604 C.ZA.
491 C.ZA.
171 C.ZA.
109 C.ZA.
410 C.ZA.
83 C.ZA.0
125 C.ZA.
95 C.ZA.0
96 C.ZA.0
445 C.ZA.
78 C.ZA.0
577 C.ZA.
432 C.ZA.
293 C.ZA.
110 C.ZA.
170 C.ZA.
169 C.ZA.
416 C.ZA.
45 C.ZA.0
310 C.ZA.
661 C.ZA.
304 C.ZA.
430 C.ZA.
80 C.ZA.0
573 C.ZA.
72 C.ZA.0
523 C.ZA.
167 C.ZA.
489 C.ZA.
73 C.ZA.0
462 C.ZA.
450 C.ZA.
74 C.ZA.0
292 C.ZA.
467 C.ZA.
459 C.ZA.
290 C.ZA.
294 C.ZA.
455 C.ZA.
65 C.ZA.0
442 C.ZA.
68 C.ZA.0
640 C.ZA.
127 C.ZA.
165 C.ZA.
385 C.ZA.
447 C.ZA.
482 C.ZA.
278 C.ZA.
492 C.ZA.
439 C.ZA.
471 C.ZA.
608 C.ZA.
448 C.ZA.
47 C.ZA.0
50 C.ZA.0
132 C.ZA.
421 C.ZA.
77 C.ZA.0
417 C.ZA.
168 C.ZA.
474 C.ZA.
494 C.ZA.
88 C.ZA.0
622 C.ZA.
133 C.ZA.
52 C.ZA.0
82 C.ZA.0
106 C.ZA.
563 C.ZA.
118 C.TZ.
466 C.ZA.
148 C.ZA.
423 C.ZA.
665 C.ZW.
666 C.ZW.
261 C.MW.
568 C.ZA.
641 C.ZM.
43 C.ZA.0
276 C.ZA.
193 07 BC
569 C.ZA.
143 C.ZA.
473 C.ZA.
574 C.ZA.
487 C.ZA.
557 C.ZA.
176 C.ZA.
267 C.ZA.
521 C.ZA.
321 C.ZA.
326 C.ZA.
606 C.ZA.
158 C.ZA.
449 C.ZA.
105 C.ZA.
116 C.TZ.
656 C.ZA.
120 C.ZA.
438 C.ZA.
561 C.ZA.
137 C.ZA.
452 C.ZA.
238 C.MW.
371 C.BW.
372 C.BW.
374 C.BW.
520 C.ZA.
382 C.KE.
373 C.BW.

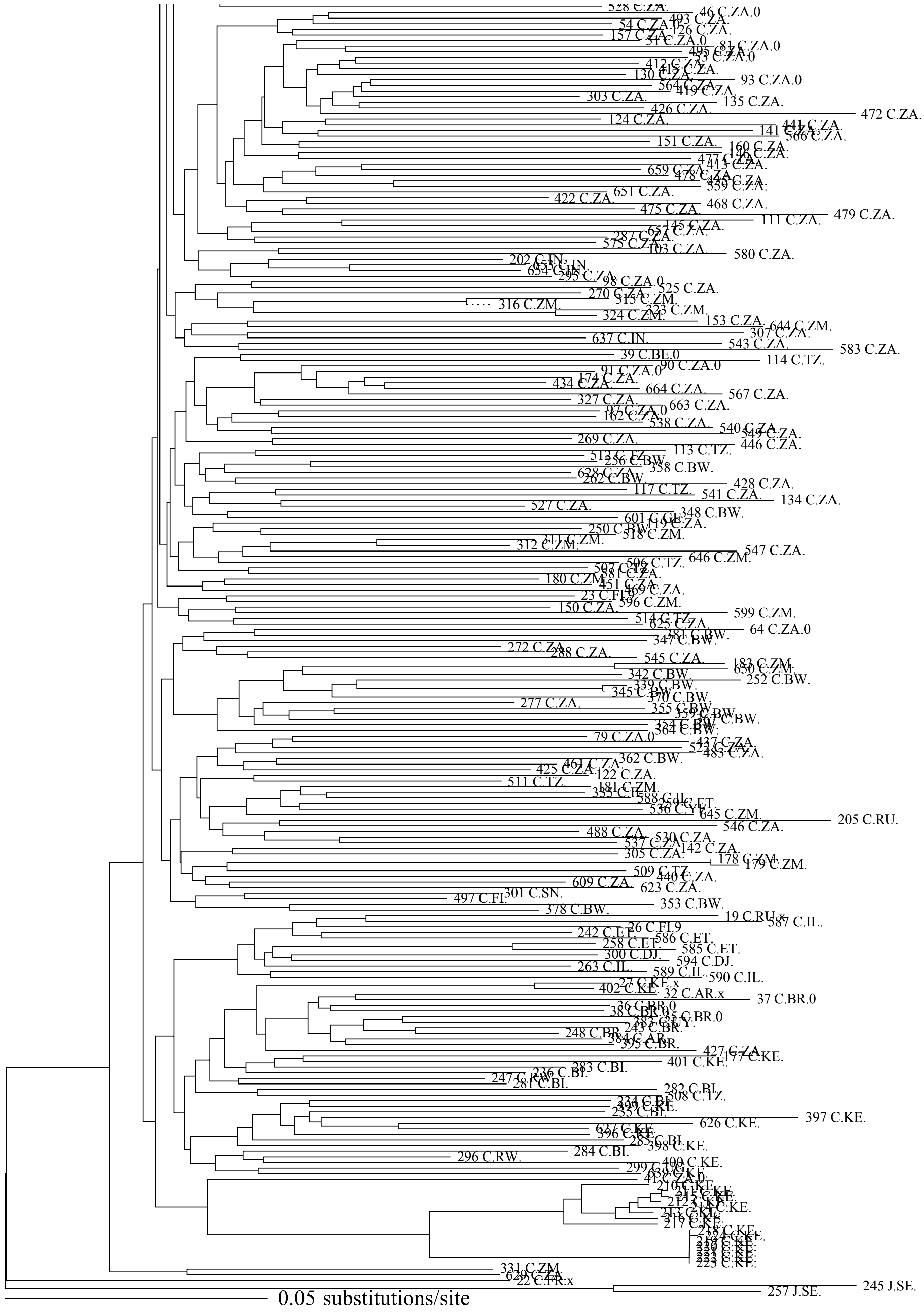
0.05 substitutions/site

Suppl. figure 3. The breakpoint frequency of CRF12, CRF28, and CRF29 sequences.

The breakpoint frequency = N/M, where N = the total number of BF recombinants that meet the following criteria: (1) breakpoints are within group breakpoint median value ± 98nt, (2) the same subtypes flanking the breakpoint as in the CRF group; M = the total number of BF recombinants that span this genomic region. Solid arrow: breakpoints at fixed positions; open arrow: breakpoint region.

CRF12 group

Breakpoint number	1	2	3	4	5	6	7
Genome graph	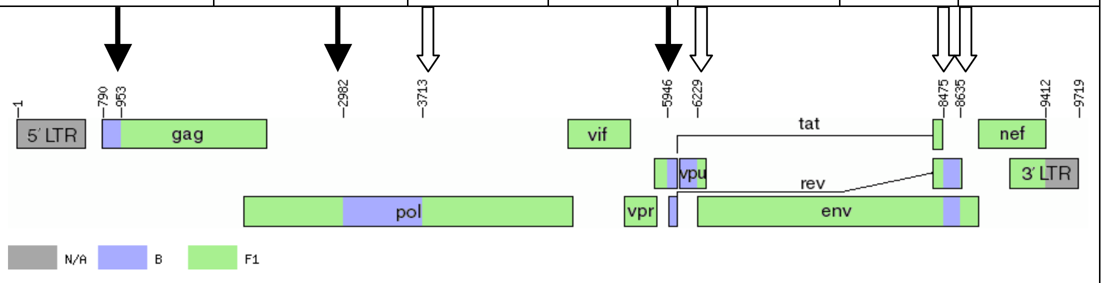						
Breakpoint locations Of this CRF	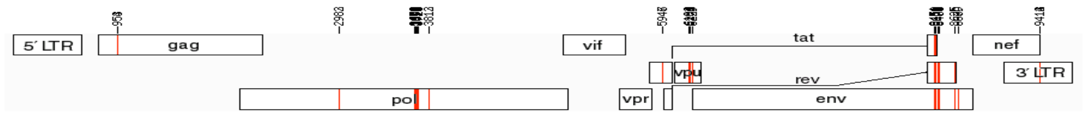						
Breakpoint range	953	2982	3679-3812	5946	6193-6229	8450-8485	8635-8669
Breakpoint median	953	2982	3713	5946	6229	8475	8635
Interquartile range (mean)	const. (953)	const. (2982)	3692-3713 (3722)	const. (5946)	6200-6229 (6216)	8475-8484 (8474)	8635-8669 (8649)
# of complete BF recombinants	Sequence total: 56 (Argentina: 22, Brazil: 23)						
Frequency of full-length BF recombinants that bear bk within median ± 98nt (world)	20/56	13/56	30/56	27/56	24/56	26/56	25/56
Frequency of full-length BF recombinants that bear bk within median ± 98nt (Brazil)	0/23	1/23	8/23	1/23	0/23	0/23	2/23
Frequency of full-length BF recombinants that bear bk within median ± 98nt (Argentina)	15/22	10/22	15/22	18/22	15/22	17/22	15/22
# of SouthAmerica BF fragments	Sequence total: 751 (Argentina: 639, Brazil: 109)						
Frequency of fragmental BF recombinants that bear bk within median ± 98nt (S. America)	0/2	333/685	11/11	0/0	25/28	4/5	1/5
Frequency of fragmental BF recombinants that bear bk within median ± 98nt (Brazil)	0/2	11/80	0/0	0/0	0/0	4/5	1/5
Frequency of fragmental BF recombinants that bear bk within median ± 98nt (Argentina)	0/0	322/603	11/11	0/0	25/28	0/0	0/0

CRF28 group:

Breakpoint number	1	2	
Genome graph	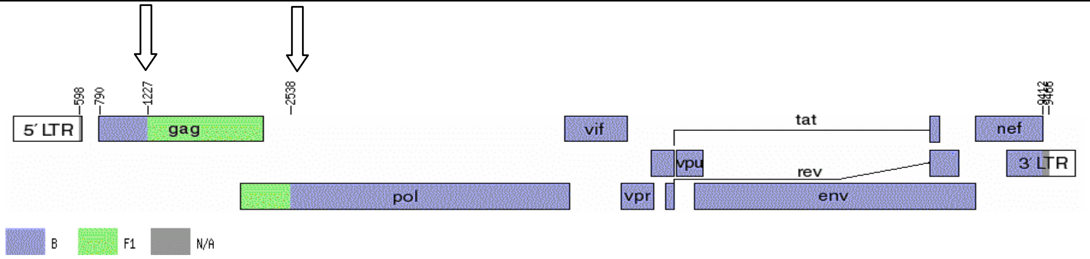		
Breakpoint locations Of this CRF	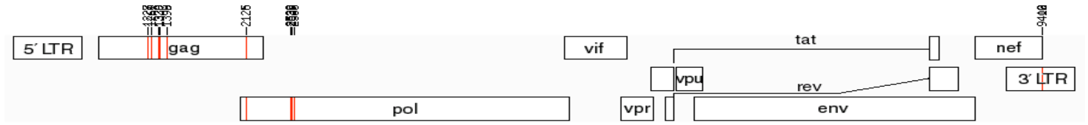		
Breakpoint range	1227-1398	2538-2565	
Breakpoint median	1329	2538	
Interquartile (mean)	1278-1364 (1318)	2538-2552 (2547)	
# of complete BF recombinants	Sequence total :56 (Argentina: 22, Brazil: 23)		
Frequency of full-length BF recombinants that bear bk within median ± 98nt (world)	15/56	29/56	
Frequency of full-length BF recombinants that bear bk within median ± 98nt (Brazil)	10/23	10/23	
Frequency of full-length BF recombinants that bear bk within median ± 98nt (Argentina)	2/22	10/22	
# of SouthAmerica BF fragments	Sequence total: 751 (Argentina: 639, Brazil:109)		
Frequency of fragmental BF recombinants that bear bk within median ± 98nt (S. America)	6/9	313/621	
Frequency of fragmental BF recombinants that bear bk within median ± 98nt (Brazil)	3/4	38/61	
Frequency of fragmental BF recombinants that bear bk within median ± 98nt (Argentina)	3/5	273/558	

CRF29 group

**Breakpoint number**	**1**	**2**	**3**	**4**	
**Genome graph**	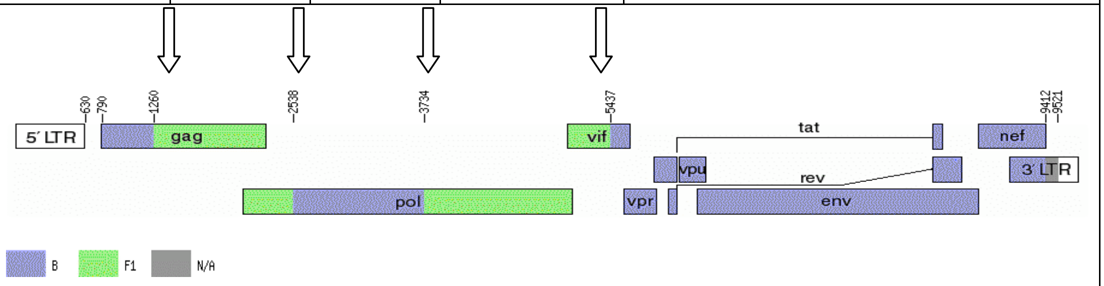				
**Breakpoint locations Of this CRF**	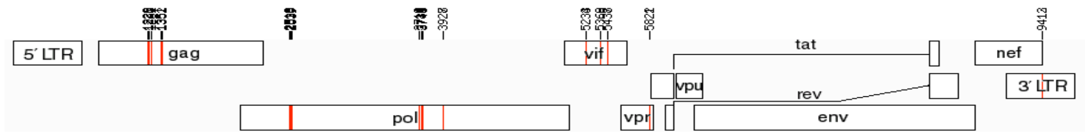				
**Breakpoint range**	1236-1351	2518-2538	3734-3927	5233-5437	
**Breakpoint median**	1260	2538	3746	5368	
**Interquartile (mean)**	1248-1306 (1282)	2528-2538 (2531)	3740-3837 (3802)	5301-5403 (5346)	
**# of complete BF recombinants**	Sequence total :56 (Argentina: 22, Brazil: 23)				
**Frequency of full-length BF recombinants that bear bk within median ± 98nt (world)**	16/56	29/56	30/56	7/56	
**Frequency of full-length BF recombinants that bear bk within median ± 98nt (Brazil)**	8/23	10/23	8/23	6/23	
**Frequency of full-length BF recombinants that bear bk within median ± 98nt (Argentina)**	4/22	10/22	15/22	1/22	
**# of SouthAmerica BF fragments**	Sequence total: 751 (Argentina: 639, Brazil: 109)				
**Frequency of fragmental BF recombinants that bear bk within median ± 98nt (S. America)**	4/9	313/621	11/11	0/0	
**Frequency of fragmental BF recombinants that bear bk within median ± 98nt (Brazil)**	1/4	38/61	0/0	0/0	
**Frequency of fragmental BF recombinants that bear bk within median ± 98nt (Argentina)**	3/5	273/558	11/11	0/0	
